# Why not both? Rethinking categorical and continuous approaches to bilingualism

**DOI:** 10.1177/13670069211031986

**Published:** 2021-07-16

**Authors:** Lena V. Kremin, Krista Byers-Heinlein

**Affiliations:** Concordia University, Canada

**Keywords:** Bilingualism, latent variable models, factor mixture model, grade-of-membership model

## Abstract

**Aims and Objectives:**

Bilingualism is a complex construct, and it can be difficult to define and model. This paper proposes that the field of bilingualism can draw from other fields of psychology, by integrating advanced psychometric models that incorporate both categorical and continuous properties. These models can unify the widespread use of bilingual and monolingual groups that exist in the literature with recent proposals that bilingualism should be viewed as a continuous variable.

**Approach:**

In the paper, we highlight two models of potential interest: the factor mixture model and the grade-of-membership model. These models simultaneously allow for the formation of different categories of speakers and for continuous variation to exist within these categories. We discuss how these models could be implemented in bilingualism research, including how to develop these models. When using either of the two models, researchers can conduct their analyses on either the categorical or continuous information, or a combination of the two, depending on which is most appropriate to address their research question.

**Conclusions:**

The field of bilingualism research could benefit from incorporating more complex models into definitions of bilingualism. To help various subfields of bilingualism research converge on appropriate models, we encourage researchers to pre-register their model selection and planned analyses, as well as to share their data and analysis scripts.

**Originality:**

The paper uniquely proposes the incorporation of advanced statistical psychometric methods for defining and modeling bilingualism.

**Significance:**

Conceptualizing bilingualism within the context of these more flexible models will allow a wide variety of research questions to be addressed. Ultimately, this will help to advance theory and lead to a fuller and deeper understanding of bilingualism.

## Introduction

Bilingualism is a complex construct that has been redefined over the past several decades. Scholars once defined bilinguals exclusively as a small group of speakers who were perfectly “balanced” in both of their languages (Lambert et al., 1959). The definition of bilingualism has since expanded to include speakers with varying degrees of proficiency and different language experiences. This change is reflected by more than 100 different group labels for bilinguals identified in the literature, such as “fully bilingual,” “English Language Learners,” and “successive bilingual Turkish-speaking children” (Surrain & Luk, 2017). As the definition of bilingualism evolves, models of bilingualism and the corresponding statistical techniques must develop as well. Traditionally, researchers have used a categorical approach to conceptualize bilingualism with analyses focused on the comparison of discrete groups of individuals (e.g., monolinguals and bilinguals). However, recent proposals in the literature suggest that instead of creating discrete groups, bilingualism should be modeled and analyzed as a continuous construct (e.g., Baum & Titone, 2014; de Bruin, 2019; Luk & Bialystok, 2013). This proposal has important consequences for how bilingualism is conceptualized in theory and how data are analyzed, but should bilingualism researchers abandon a categorical approach entirely? Are there ways for bilingualism to be defined and modeled beyond strictly categorical or continuous approaches? Drawing from recent advances in psychometrics and latent variable models, this paper introduces models that integrate both categorical and continuous properties and then discusses how researchers can use these models to address complex questions in the field of bilingualism.

## Current models and definitions of bilingualism

An individual’s bilingual status is not a trait that can be directly measured: bilingualism cannot be determined in the same way as someone’s height, for example. In the psychometrics literature, a construct such as bilingualism that can only be measured indirectly and is theoretical in nature is referred to as a latent construct. When measuring bilingualism, researchers often rely on a combination of observable indicators, such as language proficiency and exposure to determine an individual’s bilingual status (Anderson et al., 2018; Li et al., 2006, 2014; Marian et al., 2007; Marian & Hayakawa, 2020). The use of multiple measures when evaluating an individual’s bilingual status indicates that researchers (at least implicitly) view bilingualism as a multidimensional construct, or a construct comprised of “a number of interrelated attributes or dimensions” (Law et al., 1998, p. 741). Given that the construct of bilingualism is both latent and multidimensional, deciding how to combine multiple, observable measures into one parsimonious model is a crucial step in theory development and data analysis. Multidimensional constructs most frequently follow either categorical or continuous models, depending on the theoretical relation between a latent construct and its observable measures (Diamantopoulos et al., 2008; Law et al., 1998; Meehl, 1995; Polites et al., 2012; Waller & Meehl, 1998). In the field of bilingualism, researchers frequently use a categorical model, but more researchers are turning to continuous approaches based on recent theoretical perspectives.

### Categorical model

**Figure 1.**
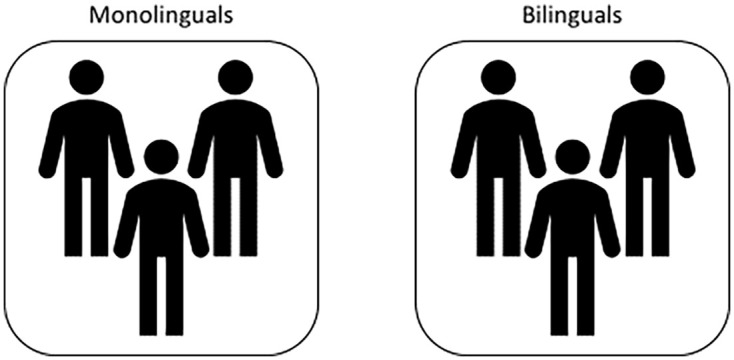
Representation of a categorical model of bilingualism.

Much of the early literature on bilingualism followed a categorical model and compared bilinguals and monolinguals as discrete groups (see Figure 1). For example, a seminal study by Peal and Lambert (1962) compared “balanced” bilingual and monolingual children on several measures of intelligence and achievement, and the results dispelled the myth that bilingualism was detrimental to children’s development. In another classic study, Ianco-Worrall (1972) found that bilingual children, defined as those who were exposed to two languages regularly and who demonstrated competence in those languages, realize the arbitrary nature of the mapping from a word’s sound to its meaning earlier than monolinguals, suggesting bilinguals have advanced semantic knowledge. The comparison of bilinguals and monolinguals has also been used in more contemporary research, and a large number of studies have found differences in group comparisons of monolinguals and bilinguals, across cognitive (Bialystok, 2004; Costa et al., 2009; Prior & Macwhinney, 2010; Zirnstein et al., 2018), neuroscientific (see Del Maschio & Abutalebi, 2019; Pliatsikas & Schweiter, 2019 for reviews), and linguistic domains (e.g., Byers-Heinlein et al., 2010; Kaushanskaya & Marian, 2009; Sebastián-Gallés et al., 2012), among many other subfields of study.

**Figure 2.**
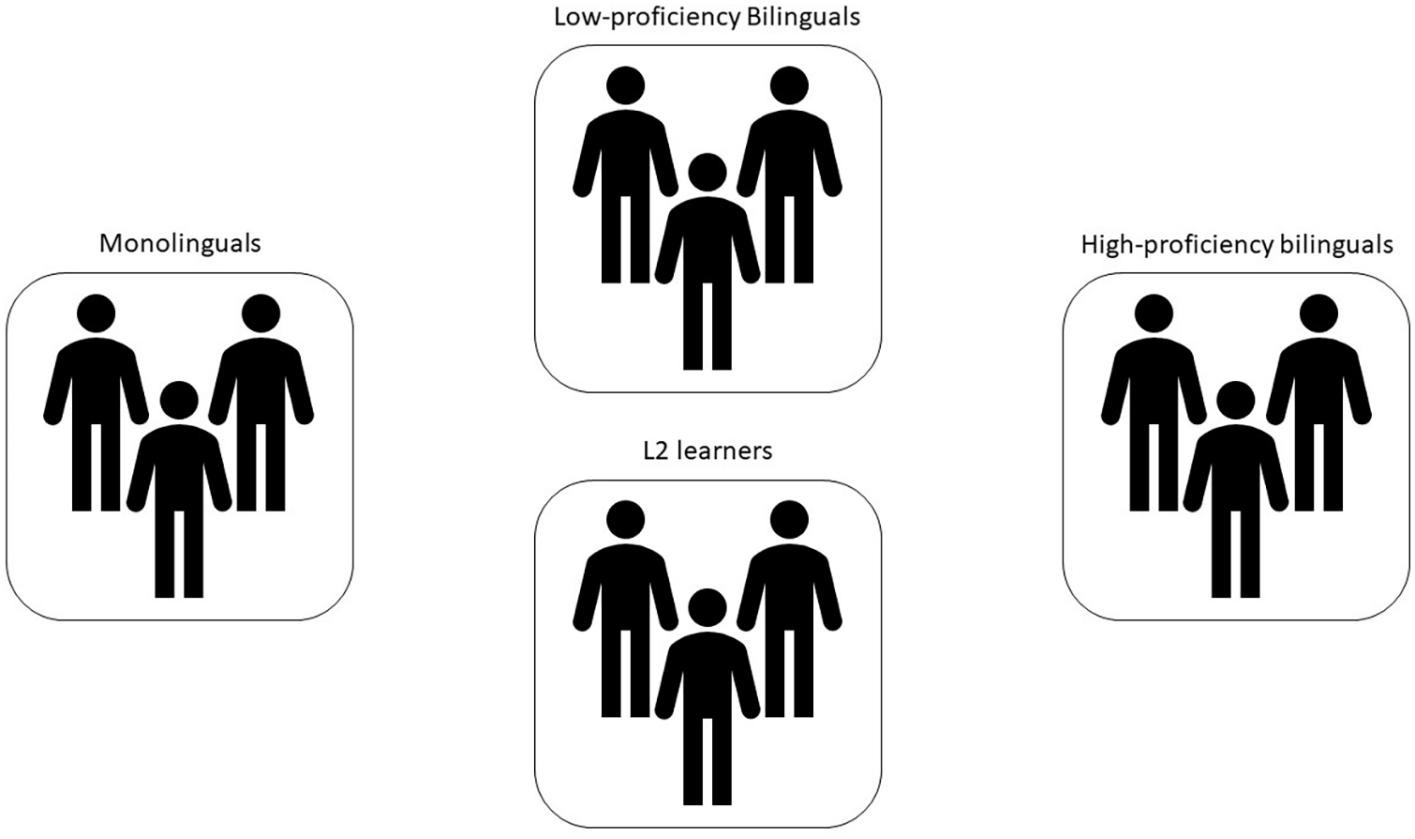
Representation of a categorical model of bilingualism with many different possible groups of bilinguals.

When these bilingual and monolingual groups are examined more closely, however, variation within each group becomes apparent. For instance, bilinguals may have different ages of acquisition, language combinations, and/or degrees of proficiency, and monolinguals may have different amounts of exposure to a second language (L2) across their lifespan (e.g., many researchers consider adults to be monolingual even if they had some foreign language education in school). Researchers have recognized that the heterogeneity within the traditionally-defined bilingual and monolingual groups could obscure differences in performance within each of these groups (e.g., Abutalebi & Rietbergen, 2014; Baum & Titone, 2014; de Bruin, 2019; DeLuca et al., 2019; Luk, 2015; MacCallum et al., 2002). In order to accommodate the variation within groups and gain a deeper understanding of bilingualism, many researchers use more nuanced bilingual groups, such as “early bilinguals,” “French–English bilinguals,” and “nearly balanced bilinguals” (see Figure 2; Surrain & Luk, 2017). With the increased number of bilingual groups, researchers can compare different groups of bilinguals to each other. This allows a categorical model of bilingualism to be used to address a wide variety of research questions across subfields of bilingualism research, from infancy (e.g., Bosch & Sebastián-Gallés, 1997) to older adulthood (e.g., Bialystok, 2004), addressing questions ranging from language acquisition (e.g., Müller & Hulk, 2001) to cognitive benefits (e.g., Costa et al., 2009). This practice has allowed for a wide variety of comparisons to be made between bilinguals and monolinguals, as well as between different types of bilinguals, and has generated a large amount of knowledge about bilingualism.

While increasing the number of bilingual categories better captures the variability in bilinguals’ experiences and abilities, categories are often poorly defined in research articles, limiting the interpretability of results (de Bruin, 2019; Hulstijn, 2012; Lehtonen et al., 2018; Surrain & Luk, 2017). This lack of clarity can be attributed to the wide variety of measures used to categorize participants and arbitrary cutoffs that may differ from study to study. Currently, there are many ways that researchers evaluate an individual’s bilingual status. For example, there are several different questionnaires available to assess an individual’s language background, some of which were designed for use with adult samples (Language and Social Background Questionnaire, Anderson et al., 2018; Language History Questionnaire (LHQ), Li et al., 2006, 2014; Language Experience and Proficiency Questionnaire (LEAP-Q), Marian et al., 2007), while others were designed for use with infant and/or child samples (Language Exposure Questionnaire, Bosch & Sebastián-Gallés, 2001; Multilingual Approach to Parent Language Estimates, Byers-Heinlein et al., 2020; Language Exposure Assessment Tool, DeAnda et al., 2016; Alberta Language Development Questionnaire, Paradis et al., 2010; Bilingual Language Experience Calculator, Unsworth, 2013). While these questionnaires have similar measures, they are not identical. It would therefore be hypothetically possible that an individual could be placed into a different language group based on which questionnaire is used. Even if the same questionnaire is used across studies, the information gathered may not be used in the same way if each study prioritizes different components of a questionnaire (e.g., focusing on age of acquisition vs. frequency of use in the home).

Additionally, groups are often formed based on different cutoffs (often due to the nature of the sample available), which have varying levels of empirical support. For example, a single study may compare a group of early-bilinguals and late-bilinguals, but the definition of who qualifies as an early-bilingual versus a late-bilingual may vary across studies. To illustrate, Tao and colleagues (2011) placed bilinguals into the early or late group if their L2 exposure began before the age of 6 years or after the age of 12 years (respectively), whereas Baker and Trofimovich (2005) placed bilinguals into the early or late group if their L2 exposure began before the age of 13 years or after the age of 15 years (respectively). Therefore, even if studies use the same labels for their bilingual groups, the groups may have different characteristics, making it difficult to synthesize findings. Because researchers cannot rely on the particular labels used in one study when comparing across multiple studies, extensive details on the bilingual sample(s) in a given study are necessary for results to be interpreted within the context of the literature.

In addition to being difficult to synthesize across studies, categorizing participants into discrete groups could have unintended consequences for statistical analyses and replicability. First, conducting group analyses when the variable of interest is actually continuous reduces statistical power and increases the chance of a Type I error (Altman & Royston, 2006; Cohen, 1983). Second, categorization could limit the reproducibility of the results if groups are formed based on an individual sample (e.g., median split), as the groups would then be quantitatively different across studies (Altman & Royston, 2006). Lastly, if groups are formed based on values of a continuous measure, a large amount of information and variability from that measure can be lost when such groups are formed (MacCallum et al., 2002). For example, if a sample of bilinguals is split based on participants’ age of acquisition, there will be “early” and “late” learners. This reduces the variability within age of acquisition, and the individual ages for each participant are effectively lost. Moreover, if the split is made at an arbitrary cutoff point (say the median age of acquisition of 10 years), then those with an age of acquisition of 9 and 11 years are placed in different groups even though they may be more similar to each other than to other members of their group (i.e., an age of acquisition of 9 years is more similar to that of 11 years than that of 1 year; Altman & Royston, 2006; MacCallum et al., 2002). In sum, dividing bilinguals into groups when the underlying construct is continuous has statistical consequences and could obscure our understanding of bilingualism.

### Continuous model

**Figure 3.**
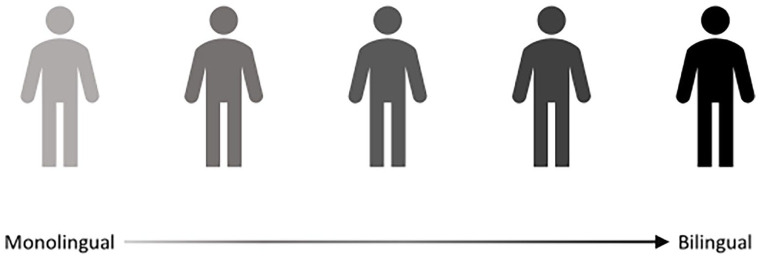
Representation of a continuous model of bilingualism.

In order to account for the full spectrum of bilinguals’ experiences and abilities, some scholars have proposed that bilingualism should be viewed and analyzed as a continuous variable (Baum & Titone, 2014; de Bruin, 2019; Kaushanskaya & Prior, 2015; Marian & Hayakawa, 2020; Takahesu Tabori et al., 2018). Under such an approach, the continuum would span the range from completely monolingual (i.e., never having any exposure to a L2) to fully proficient bilingual (i.e., “balanced;” see Figure 3). It would be possible to create a continuum of bilingualism based on a single variable (e.g., years spent speaking two languages). However, given that bilingualism is a latent and multidimensional construct, using a variety of measures might better place individuals on a bilingualism continuum. These different measures will need to be mathematically combined into a final bilingualism score. For example, the concept of language entropy incorporates participants’ responses to questions about their language exposure, language proficiency, language use in different contexts, and L2 accent perception on a single continuous scale (Gullifer & Titone, 2020). When using a continuous approach, scholars will need to determine which measures to include and how they will be algebraically combined to result in a final bilingualism score (Law et al., 1998), for example giving more weight to some dimensions (e.g., age of acquisition) than others (e.g., time spent listening to the radio in the L2). Marian and Hayakawa (2020) have recently dubbed this type of standardized bilingualism index a “Bilingualism Quotient.” It is important to note that the relationship between different measures and the final bilingualism score does not need to be linear. For instance, age of acquisition could follow a pattern of non-linear decrease resembling threshold effects seen in sensitive periods for language acquisition (Werker & Hensch, 2015; Werker & Tees, 2005).

A continuous model would allow researchers to investigate subtle effects of bilingualism and would therefore be useful in specialized applications. For instance, the investigation of potential cognitive benefits of bilingualism in adults could benefit from the ability to detect smaller effects, and using a continuous model could potentially establish thresholds to see effects of bilingualism in this domain (e.g., Cummins, 1976; De Cat et al., 2018; Ricciardelli, 1992). While using a continuous model for bilingualism may be appropriate in some research domains, it is unlikely that this model will become the standard across all subfields of bilingual research, as the benefits may not apply to certain subfield-specific contexts. For example, some subfields (e.g., research with special populations such as infants, or children with developmental delays) will tend to focus on large effects in smaller samples, making a continuous model less practical than a categorical approach. Moreover, categorical approaches might be more appropriate than continuous ones in some research contexts, for example enrollment in a language immersion program is inherently categorical (i.e., children are or are not enrolled), a point that we will return to later in this paper. Therefore, both continuous and categorical models may be useful in advancing bilingualism research depending on the particular study.

## Expanding models of bilingualism

Both categorical and continuous models of bilingualism have their advantages and disadvantages. Categorical models are easy to interpret, but the groups used in the analyses may be heterogenous. Continuous models accommodate more individual variation but may not be practical in all bilingualism research and may be inappropriate if the underlying construct is actually discontinuous. Each one can answer different research questions, but given that bilingualism is a complex construct, some research questions may be best addressed by some combination of the two. Are models available that better reflect the complexity of bilingualism by incorporating the advantages of both categorical and continuous models? Other areas of research, such as psychometrics, may offer innovative solutions to defining and modeling bilingualism (Borsboom et al., 2016). While there are many different psychometric models that bilingualism researchers can consider, here we introduce two interesting possibilities: the factor mixture model; and the grade-of-membership model. Like current approaches to modeling bilingualism that rely on participants’ responses to a series of questionnaires or tasks, both of these models find patterns within participants’ responses about their language history, proficiency, and any other variables relevant to defining bilingualism (Andreotti et al., 2009; Clark et al., 2013; Masyn et al., 2010). Additionally, researchers can decide which participant data are of theoretical interest to include in the model (e.g., language attitudes, proficiency, and age of exposure). Unlike current approaches, categories are not pre-defined by the researcher, nor are they formed by potentially arbitrary cutoffs determined by the researcher. Instead, categories emerge as clusters based on statistical patterns in the data. Furthermore, each of these models offers the possibility of analyzing data continuously, which could increase statistical power of analyses involving the dependent variable if bilingualism does exist on a continuum (Altman & Royston, 2006; Cohen, 1983). In sum, each of these models is more comprehensive than current research practices and would allow researchers to incorporate both categorical and continuous properties when analyzing their data.

### Factor mixture model

Factor mixture models are based on the idea that variation can exist within categories (Lubke & Muthén, 2005; McLachlan & Peel, 2004), thus individuals are both placed into separate categories and given a score on a continuous scale (Clark et al., 2013). Depending on the constraints set when developing the model, this continuous score could be interpretable relative to all participants, or only relative to participants within the same category. For an example unrelated to bilingualism, children could be divided into categories based on whether or not they have a conduct disorder, and the degree to which they exhibit symptoms is allowed to vary within each group (i.e., children in the group with conduct disorders vary in severity of symptoms; Clark et al., 2013).

**Figure 4.**
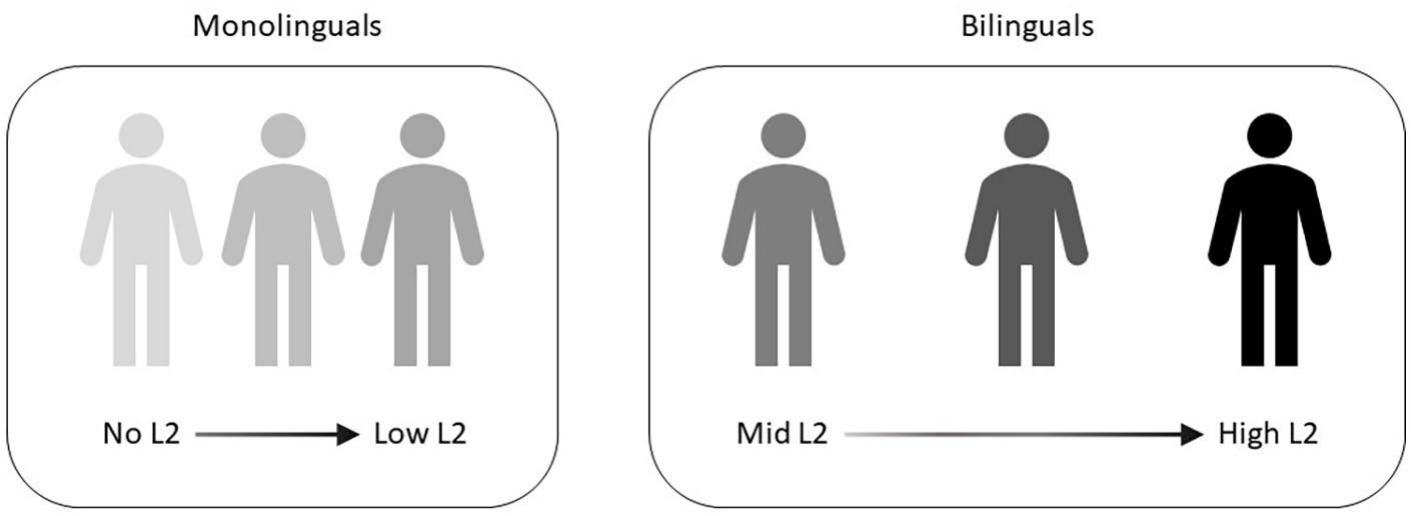
Representation of a factor fixture model of bilingualism where data can be analyzed based on categorical membership or placement on a continuum.

With the definition of bilingualism expanding beyond the view that only individuals who are “balanced” in both of their languages are bilingual, there is inherently more variation across individuals who would now be considered bilingual. Factor mixture models could capture the variation within bilinguals by classifying participants into either a monolingual or bilingual group and accounting for variation within each of those groups (see Figure 4). Factor mixture models can also accommodate multiple groups. Allowing multiple bilingual groups in a factor mixture model could potentially mirror groups that already exist in the literature (e.g., simultaneous, sequential, etc.), and subsequently capture the heterogeneity within those groups (Clark et al., 2013; Sulpizio et al., 2020). While theory can drive the number of categories and the measures that are included in a final bilingualism score, it should be noted that the number of groups and the way that different variables contribute to the continuous score are typically determined through an iterative modeling process. In this process, the number of groups and how different variables define group membership are systematically varied to find the strongest factor mixture model, although the researcher can set theoretically-motivated constraints on models that will be considered (Clark et al., 2013; Nylund et al., 2007).

For a concrete example, imagine Dr Factor-Mixture who is working on a project investigating the potential effect of bilingualism on a memory task and plans to use a factor mixture model to identify bilinguals and monolinguals in her research. Dr Factor-Mixture collects information from 150 participants – the minimum recommended sample size for creating a factor mixture model (Lubke & Neale, 2006) – about their language experience and history via the LHQ (Li et al., 2014) before they complete the memory task. Once all her data are collected, she uses the participants’ responses to the questionnaire to determine their bilingual status. She will use the FactMixtAnalysis package (Viroli, 2012) in R, her preferred statistical software (although she could have also used Mplus; Muthén & Muthén, 2016). Using the observed patterns of responses to the questionnaire, participants are placed into different groups and within each group are given a composite, final score on a continuous scale indicating how they are situated within the group (Clark et al., 2013; DiStefano et al., 2009). Dr Factor-Mixture can choose a specific type of factor mixture model that either uses the same or different variables to determine continuous scores in each group depending on her research goals and theoretical conceptualization of bilingualism (Clark et al., 2013). Dr Factor-Mixture expects that there may be different types of bilinguals in her sample (i.e., sequential and simultaneous bilinguals), so she runs models with different numbers of expected groups. In order to compare the goodness of fit for different models and identify the most parsimonious model, Dr Factor-Mixture compares the Akaike information criterion and Bayesian information criterion values of each model and selects the one with the lowest value (Hallquist & Wright, 2014). These values indicate how closely the data fit a particular model. When comparing the results, the model that contains four groups built from different variables for each group is the most parsimonious and is selected as the final model. When Dr Factor-Mixture examines the output of the final model, she looks at how different variables contribute to group membership and sees that these groups could be described as monolingual, sequential low-proficiency bilingual, sequential high-proficiency bilingual, and simultaneous high-proficiency bilingual. Dr Factor-Mixture can now analyze the participants’ scores from the memory task categorically using the groups identified in the model in an anaylysis of variance (ANOVA) or use a regression model to additionally incorporate participants’ continuous scores within each group.

### Grade-of-membership models

Grade-of-membership models also allow for variation within categories. Such models place individuals into different categories, but uniquely allow for individuals to simultaneously belong to different categories to varying degrees (Andreotti et al., 2009; Erosheva, 2005). Some individuals overwhelmingly belong to one group, and the model consequently places them into that group. Some individuals may be somewhere in between multiple groups, belonging to different groups to different degrees. Grade-of-membership models capture in-between cases, where individuals’ categorization is not as clear, through a “fuzzy set.” This set has no definitive boundaries, and individuals belong to this set to different degrees. Grade-of-membership models can accommodate multiple groups and the overlap between them. For an example unrelated to bilingualism, individuals can simultaneously be affiliated with different political parties, because their ideologies fall somewhere in between those most characteristic of the different groups (Gormley & Murphy, 2009).

**Figure 5.**
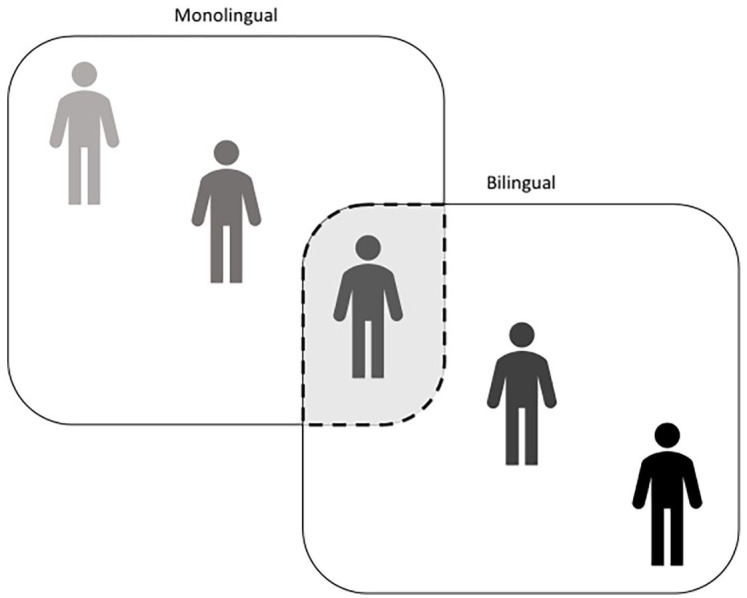
Representation of a grade-of-membership model of bilingualism where data can be analyzed based on categorical membership or placement on a continuum.

When applied to bilingualism, a grade-of-membership model could still include monolingual and bilingual groups but would also accommodate individuals who do not necessarily fit strict definitions for either group (see Figure 5). Imagine an individual who studied a L2 for several years and obtained an intermediate level of proficiency, but who no longer uses the language frequently. They might not qualify as either monolingual or bilingual by the definitions used in many studies. Individuals such as this have often been less studied in the literature. However, it might still be important to include these individuals in studies in order to gain a more comprehensive view of bilingualism. Therefore, incorporating a grade-of-membership model and the “fuzzy set” between different groups of bilinguals and monolinguals could offer more insight into how language experience influences a wide variety of factors.

To see this in practice, imagine Dr Grade-O’Membership who is investigating the effect of bilingualism on word learning in adults. Dr Grade-O’Membership recruited 200 participants – the minimum recommended sample size to allow for accurate group identification in grade-of-membership models (Holmes Finch, 2021) – and asked his participants extensive questions about their language history and proficiency using the LEAP-Q (Marian et al., 2007). He decides to analyze the responses to these questions using Mplus (Asparouhov & Muthen, 2006; Muthén & Muthén, 2016), but he could have also used the mixedMem package in R (Wang & Erosheva, 2015). Dr Grade-O’Membership builds several models with different numbers of groups and selects the final model, which happens to have only two groups, by identifying the model with the lowest truncated sum of squared Pearson residuals (*Χ*^2^_*tr*_; Erosheva et al., 2007; Holmes Finch, 2021). Based on their responses, each participant is given a probability of belonging to each of the two groups identified in the sample; the total of these probabilities will sum to one. Dr Grade-O’Membership can determine if a participant should be placed in the bilingual or monolingual group, based on the group that the model says they have the highest probability of belonging to. He notices that very few participants have intermediate probabilities, so decides that his sample has more of a categorical structure. He then determines which group learned more words using a two-sample *t*-test. He could also use the probability that each participant belongs to the bilingual group to analyze the data continuously and examine the relationship between the degree of bilingualism and the number of words learned using a regression model.

### Incorporation of new models

Both the factor mixture model and the grade-of-membership model are tools that researchers can use to better represent the underlying structure of bilingualism and better address questions in the field. They could be incorporated into research on bilingualism by following several steps. In order to benefit from either of these comprehensive model approaches, a new model will first need to be created and validated following the steps explained in the hypothetical examples above. This would involve creating new datasets or using pre-existing databases with information about a wide range of bilinguals and monolinguals on a variety of bilingualism measures, such as language proficiency and history (e.g., via an extensive questionnaire such as the LHQ (Li et al., 2014); or LEAP-Q (Marian et al., 2007)). Then various iterations of either the factor mixture model or grade-of-membership model would be built and evaluated for goodness of fit using statistical software (Clark et al., 2013). Once a parsimonious model has been fit to the data, researchers can use the model to address a variety of research questions. Researchers can use models that they have built themselves or models built by other researchers. If several studies addressing the same research question use the same model, researchers will be able to make direct comparisons across these studies.

For an example of how researchers could use previous models, take Dr Resourceful who is studying attention. Dr Resourceful is only able to test 75 participants, which is not an adequate sample size to develop their own factor mixture model or grade-of-membership model. Instead, they opt to use the model developed by Dr Factor-Mixture to evaluate the bilingual status of the participants they do have, because they are studying a similar population. Dr Resourceful will need to give their participants the LHQ (Li et al., 2014), so that participants answer the same questions that Dr Factor-Mixture used to create the model, and feed participants’ responses to specific items into the model. This will output a bilingualism score for each participant, as well as identifying which of the four groups from the original model the participant belongs to. Dr Resourceful discovers that none of their participants are placed into the sequential high-proficiency group but are split relatively equally into the remaining groups. Because each of the groups has different variables contributing the bilingualism score (due to the nature of the original model developed by Dr Factor-Mixture), a continuous analysis of all participants is not possible in this model, but Dr Resourceful can approach their analyses in one of two ways. They can analyze the data through a categorical lens, using the monolingual, sequential low-proficiency, and simultaneous high-proficiency groups formed by the model, or they can incorporate both the categorical and continuous information from the model in the analyses by computing a separate regression model using the final bilingualism score for each of their groups.

The factor mixture model and grade-of-membership model are simply two of many models that researchers could consider employing in the field of bilingualism. If we look to the field of psychometrics, there are a wide variety of models that could help researchers better define and model bilingualism, such as different forms of factor analysis (Anderson et al., 2018) or cluster analysis (Woodbury & Manton, 1989). In using more complex models, information on modeling decisions will need to be made explicit, and assumptions about the nature of bilingualism could ultimately be challenged. By addressing these issues in the field, researchers will be able to drive theories of bilingualism forward. While these complex models will help to operationalize bilingualism, it is necessary to address how to best incorporate them into the field.

### Standardization in the field

When moving towards more comprehensive models of bilingualism, some may argue that there is a single best model of bilingualism that should be used in the field, including across different subfields and studies (Marian & Hayakawa, 2020). However, this approach could face obstacles in the measures that are available across the stages of development and the statistical analyses that can be conducted with different populations. Additionally, standardization within the field of bilingualism could limit the number and type of research questions that can be addressed.

First, a standard definition of bilingualism may be difficult to implement across different populations and stages of development. For example, it is possible to gather a wide range of data on an adult’s language proficiency and background through questionnaires or language tests (Anderson et al., 2018; Li et al., 2014; Marian et al., 2007; McNamara, 2000). This provides a comprehensive view of an individual’s language experience that could be used in analyses. However, gathering the same in-depth information on an infant’s language experience is much more difficult. Infants are unable to respond to direct questions, so their caregivers must provide information about their language experience, which is often limited to information about their language exposure (Bosch & Sebastián-Gallés, 2001; Byers-Heinlein et al., 2020). Trying to use the same standardized measure for both adults and infants would be ineffective and ultimately unsuccessful. We argue instead that in order to increase transparency, bolster comparisons across studies, and help replication efforts, researchers should include detailed descriptions of their definition, measures, and model of bilingualism (Esposito et al., 2015; Luk et al., 2017). Furthermore, where possible, researchers who work with similar populations should try to reach a consensus on using a single measure (Cat et al., 2021).

Second, bilingualism may have a different underlying structure in different target populations or in the context of different research questions, and, as discussed above, it is important that statistical analyses accurately reflect this underlying structure (Altman & Royston, 2006; Cohen, 1983; MacCallum et al., 2002). For example, in a study investigating if there is a difference in bilinguals’ and monolinguals’ ability to discriminate two languages in infancy (Byers-Heinlein et al., 2010; Nazzi et al., 2000), a categorical construct such as language group (i.e., monolingual vs. bilingual) might appropriately characterize the sample, and *t*-tests, ANOVAs, or regressions with categorical predictors would be appropriate analytic approaches. By contrast in a study investigating how bilingual experiences (e.g., age of acquisition of their L2) affect brain function (DeLuca et al., 2019), participants might be best characterized in terms of a continuous measure of bilingualism, and correlations or regression models would be appropriate. Finally, as this paper has proposed, in many cases the sample might have both categorical and continuous characteristics, for example in a study of undergraduate students who come from diverse monolingual and bilingual backgrounds and have different language histories. Here, either a factor mixture model or grade-of-membership model could be appropriate. Because of the variety of samples and research questions in the field of bilingualism, it is important that a variety of models be accepted in the field and for researchers to carefully consider which model best addresses their population and research question.

## Future directions

This paper has discussed four different models of bilingualism that scholars have used or could use in their research. The traditional practice of using a categorical model and the recently proposed continuous model of bilingualism are the tip of the iceberg for how bilingualism can be defined and modeled. We have suggested two other types of models for bilingualism researchers to consider: the factor mixture model and the grade-of-membership model. These models extend the current thinking about how bilingualism should be defined and understood, as they incorporate both categorical and continuous aspects.

Although the aim of this paper is to encourage researchers to consider different models of bilingualism, we caution against too many models being used across the literature. We recommend that particular subfields compare the relative theoretical and practical merits and performance of different models, and carefully consider the types of participant data used to create their models (i.e., questions about language proficiency and use, vs. questions about language attitudes). Ideally, subfields will converge on the model that is most appropriate for their research questions and populations and converge on a standard approach to collect such data (e.g., a consistent questionnaire). For the researchers who are developing models, we encourage them to pre-register the steps that they will take and the comparisons that they will make to arrive at the final model, including the number of different groups and the combinations of variables they will try. Once the model has been finalized, researchers can transparently report the creation and selection process and share their scripts, so others can use the same model. Similarly, for researchers who are using previously developed models, we suggest that they consider which model to use based on their research question and the typical models used in their subfield before data analysis begins and to pre-register this choice, as well as their commitment to use the same materials that were used in the development of the model. This will reduce the chances of *p*-hacking and tinkering with group definitions until results are statistically significant or match the original hypothesis, which can increase Type I error and lead to less robust results (Simmons et al., 2011). We also encourage all researchers to share their data to increase transparency and contribute to standardization efforts. Combined, taking these steps will help a particular subfield converge upon a single model best suited to its needs. Adopting more nuanced models will ultimately allow for a wider range of research questions to be addressed and for advancement of theories of bilingualism.
